# Non-Celiac Gluten Sensitivity: A Review

**DOI:** 10.3390/medicina55060222

**Published:** 2019-05-28

**Authors:** Anna Roszkowska, Marta Pawlicka, Anna Mroczek, Kamil Bałabuszek, Barbara Nieradko-Iwanicka

**Affiliations:** 1 Medical University of Lublin, Radziwillowska 11 Street, 20-080 Lublin, Poland; martamisztal991@gmail.com (M.P.); anna.mroczek94@wp.pl (A.M.); balkam@o2.pl (K.B.); 2 Chair and Department of Hygiene, Medical University of Lublin, Radziwillowska 11 Street, 20-080 Lublin, Poland; barbaranieradkoiwanicka@umlub.pl

**Keywords:** non-celiac gluten sensitivity, irritable bowel disease, gluten, FODMAP, wheat allergy

## Abstract

*Background and objectives:* Grain food consumption is a trigger of gluten related disorders: celiac disease, non-celiac gluten sensitivity (NCGS) and wheat allergy. They demonstrate with non-specific symptoms: bloating, abdominal discomfort, diarrhea and flatulence. Aim: The aim of the review is to summarize data about pathogenesis, symptoms and criteria of NCGS, which can be helpful for physicians. *Materials and Methods:* The PubMed and Google Scholar databases were searched in January 2019 with phrases: ’non-celiac gluten sensitivity’, non-celiac gluten sensitivity’, non-celiac wheat gluten sensitivity’, non-celiac wheat gluten sensitivity’, and gluten sensitivity’. More than 1000 results were found. A total of 67 clinical trials published between 1989 and 2019 was scanned. After skimming abstracts, 66 articles were chosen for this review; including 26 clinical trials. *Results:* In 2015, Salerno Experts’ Criteria of NCGS were published. The Salerno first step is assessing the clinical response to gluten free diet (GFD) and second is measuring the effect of reintroducing gluten after a period of treatment with GFD. Several clinical trials were based on the criteria. *Conclusions:* Symptoms of NCGS are similar to other gluten-related diseases, irritable bowel syndrome and Crohn’s disease. With Salerno Experts’ Criteria of NCGS, it is possible to diagnose patients properly and give them advice about nutritional treatment.

## 1. Introduction

Wheat, rice and maize are the most commonly consumed grains worldwide. These products are rich sources of starch—the basic dietary component for the growing human population [1]. Wheat contains gluten. In 1953 Dickie, van de Kamer and Weyers published a study confirming malabsorption after wheat consumption in patients with celiac disease (CD) [2]. Nowadays, gluten intake is considered to be the trigger of gluten related disorders (GRDs). In GRD, the gluten-free diet (GFD) is principal, effective and yet the only treatment method. The gluten-free market is still rising, not only because of growing interest and public awareness of GRDs, but also due to celebrities touting this diet by for weight loss and athletes for improved performance [3], which is debatable as grains should be the main source of energy in the human diet.

## 2. Materials and Methods

Standard up-to-date criteria were followed for review of the literature data. A search for English-language articles in the PubMed database was performed. The PubMed and Google Scholar databases were searched in January 2019 with phrases: ’non-celiac gluten sensitivity’, non-celiac gluten sensitivity’, non-celiac wheat gluten sensitivity’, non-celiac wheat gluten sensitivity’, and gluten sensitivity’. More than 1000 results were found. A total of 67 clinical trials published between 1989 and 2019 was scanned. After skimming abstracts, 66 articles were chosen for this review including 26 clinical trials.

### 2.1. Gluten Related Disorders (GRDs)

The term “gluten intolerance” includes three different conditions: CD, allergy to wheat (WA) and non-celiac gluten sensitivity (NCGS) [4]. To date, CD and WA comprise for the best known and studied entities, which are mediated by immune system [1]. WA—classified as a classic food allergy is induced by wheat (not only gluten) intake that leads to type I and type IV hypersensitivity. The crucial role in WA disorder play IgE immunoglobulins [1,5]. CD is an autoimmune disease occurring in genetically susceptible individuals with HLA-DQ2 and/or HLA-DQ8 genotypes. CD is characterized by the presence of specific serological antibodies such as: anti-tissue transglutaminase (tTG) IgA, anti-endomysium IgA (EMA) and anti-deamidated gliadin peptides IgG (DPG) [1]. There were reported cases of patients with gluten sensitivity in which allergic and autoimmune mechanisms could not be identified. They were collectively described as NCGS [1]. The NCGS or “non-celiac wheat sensitivity’’ (NCWS) has been a topic of interest in recent years. This trend is associated with a large number of studies concerning the syndrome [6,7]. The term NCWS is more adequate because of components other than gluten, that may contribute to intestinal and extra-intestinal symptoms [6]. In 1980, Cooper et al. described intestinal gluten-sensitive symptoms in 8 patients in whom CD was ruled out [8]. Further studies led to the definition of NCGS. NCGS is a condition characterized by clinical and pathological manifestations, related to gluten ingestion in individuals in whom CD and WA have been excluded [1,6,9,10]. Leccioli et al. described NCGS as a multi-factor-onset disorder, perhaps temporary and preventable, associated with an unbalanced diet [11].

Interestingly, II MHC haplotype HLA-DQ2 and HLA-DQ8 typical for CD is present only in about 50% of NCGS patients [1]. The main features of GRDs are summarized in Table 1.

### 2.2. Epidemiology of Gluten Related Disorders (GRDs)

CD morbidity, based on serological results, is estimated to be 1.1% to 1.7% worldwide [12,13]. WA among children occurs with a frequency of 0.4–9% [5,14]. Due to an absence of diagnostic markers and population studies, the prevalence of NCGS is not well established [5,6]. Although studies have been conducted by several authors, this problem is still insufficiently explored. Previous data were based primarily on questionnaires for self-reported gluten sensitivity SR-GS/self-reported NCGS. According to several authors, the NCGS prevalence is from 0.6% up to 13% of the general population [15,16,17,18,19]. NCGS was reported more often among women [16,17,18], adults in the fourth decade of life [19,20] and individuals coming from urban area [18]. Among intestinal symptoms the most frequent in NCGS are: bloating, abdominal discomfort and pain, diarrhea and flatulence. The most common extra-intestinal symptoms were: tiredness, headache and anxiety [15,16,18,20]. Differentiation between NCGS and functional gastrointestinal (GI) disease—mainly irritable bowel syndrome (IBS)—may be difficult as some of the above-mentioned symptoms overlap with IBS manifestations. Van Gils et al. pointed that 37% of self-reported gluten sensitivity individuals (SR-GS) fulfilled the Rome III criteria for IBS, in contrast to 9% prevalence in the control group [18]. Similar findings were reported by Carroccio et al. IBS symptoms were reported in 44% self-reported NCWS [15]. According to research conducted by Cabrera et al., IBS, eating disorders and lactose intolerance were present more often in SR-GS individuals than in non-SR-GS group (14.3% vs. 4.7%) [16]. Herein, discussed studies indicate that SR-GS/SR-NCGS may correlate with more frequent occurrence of IBS, comparing to the general population. However, the German Society of Allergology and Clinical Immunology emphasized that the publications about NCGS suffer from certain weaknesses: absence of validated diagnostic criteria, suitable biomarkers, frequent self-diagnosis and unconfirmed etiology of reported symptoms. Thus, the prevalence of NCGS cannot be clearly established [21].

### 2.3. Gluten

Gluten is defined as a family of proteins found in grains (wheat, rye, barley, oats). It includes two main proteins: gliadin and glutenin. Also, similar proteins such as secalin in rye, harden in barley and avenues in oats contribute to the definition of ‘gluten’ [22]. Gluten proteins are characterized by high proline and glutamine content, moreover, they are resistant to proteolytic enzymes in the gastrointestinal tract. In some individuals these peptides can cross the epithelial barrier and activate immune system: trigger an allergic (WA) or autoimmune response (CD) [5]. Incomplete digestion leads to significant changes in human gut and causes intestinal or extra-intestinal symptoms. Gliadin and other gluten proteins stimulate T-cells. Some authors suggested that amylase-tripsin inhibitors (ATIs) and fermentable oligo-, di-, and mono-saccharides and polyols (FODMAPs) may be associated with NCGS [11]. Another wheat constituent, known as agglutinin-carbohydrate binding protein and exorphins seem to influence immune system and induce damage of intestinal epithelium [11,22].

### 2.4. Amylase-Tripsin Inhibitors (ATIs)

ATIs are albumin proteins found in wheat representing up to 4% of total proteins in grains [1]. They are highly resistant to intestinal proteases [1] and may induce release of pro-inflammatory cytokines from monocytes, macrophages and dendritic cells through activation of a toll-like receptor-4 in CD and NCGS patients [1,22]. ATIs may provoke activation of innate immune cells and intestinal inflammation [21]. ATIs activate immunological system through effect on toll-like receptor-4 in CD, that was confirmed in the research conducted by Junker et al. on mice deficient in TLR4 or TLR signaling [23]. Authors observed, that their mice models were protected from intestinal and systemic immune responses during oral ATIs intake [23]. Scientists also confirmed, that ATIs stimulate monocytes, macrophages and dendritic cells *in vitro* to produce IL-8, IL-12, TNF, MCP-1 and Regulated on Activation, Normal T-cell Expressed and Secreted (RANTES) [23].

### 2.5. Fermentable Oligo-, Di- and Mono-Saccharides and Polyols (FODMAPs)

**Figure 1:**
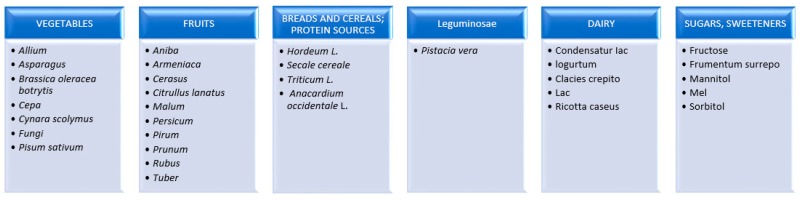
List of products being the source of fermentable oligo-, di-, and mono-saccharides and polyols (FODMAPs).

**Figure 2:**
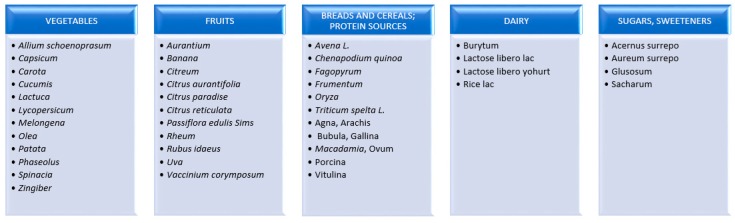
List of products low in FODMAPs.

FODMAPs are short-chain sugars with less than 10 carbon atoms in the molecule [24]. The attention of scientists in recent years was drawn to the potential contribution of FODMAPS to pathogenesis of gastrointestinal disorders [25]. The scientists from Monash University in Australia conducted thorough analysis of a group of carbohydrates, which, despite their different structures, produced similar postprandial effects. The most prevalent forms of FODMAP include: fructooligosaccharides (FOS), galactooligosaccharides (GOS), lactose, fructose, polyols, sorbitol and mannitol. Barrett et al. created a list of food products that are good sources of FODMAP (Figure 1) and poor in short chain sugars (Figure 2) [24].

Compounds belonging to the FODMAP group are not digested nor absorbed in the gastrointestinal tract. They have a strong osmotic effect and undergo rapid fermentation in the intestines, resulting in intestinal liquefaction, excessive gas production, bloating and pain. They may cause or exacerbate symptoms in susceptible patients with inflammatory bowel disease and irritable bowel syndrome (IBS) [24,25]. Numerous studies have confirmed the improvement in patients suffering from ulcerative colitis, Crohn’s Disease and IBS following the elimination of short-chain sugars from the diet [26].

Wheat is a rich source of gluten and also contains large amounts of FODMAPs, which play a key role in NCGS development [27]. Some researchers suggest that diet low in FODMAP is beneficial for NCGS patients [25].

Considering the above research results, scientists are leaning towards renaming NCGS to a more recent NCWS [27]. It should be emphasized that a diet poor in FODMAPs should not be used without medical indications, as healthy people do not benefit from such diet [24]. Moreover, it was proven that FOS and GOS, compounds belonging to FODMAPS, alike prebiotic, favor proper colonization of intestines with *Bifidobacteria* and *Lactobacilli* bacteria and limit the proliferation of *Bacteroides* spp., *Clostridium* spp. and *Escherichia coli*. There is evidence that short-chain fatty acids (SCFA)—the product of FODMAP fermentation—have protective properties against colorectal cancer [24,27]. FODMAPs are believed to have a positive effect on lipid metabolism by lowering serum cholesterol, triglycerides and phospholipids [27]. In addition, this diet leads to calcium absorption disorders, lowering its serum levels. People resigning from products that are the source of FODMAP are at risk of vitamin and antioxidants deficiency [27,28]. Therefore, it is suggested to supplement vitamins, pro- and prebiotics when switching to the low FODMAPs diet [24,27].

### 2.6. The Salerno Experts’ Criteria of NCGS

As long as the NCGS biomarker is not available, certain limitations are included in two-step diagnostic protocol introduced in 2015. However, up to date The Salerno Experts’ Criteria constitute the only accessible recommendations for diagnosis of NCGS. It should be emphasized that according to currently used criteria, NCGS should not be based only on exclusion diagnosis, which is new in comparison to the former practice [29]. Thus, the guidelines indicate the need of a standardized procedure: 6-week course of gluten-free diet—with the simultaneous, continuous assessment of symptoms and their intensity, followed by measuring the effect of reintroducing gluten after a period of treatment with GFD. A modified version of the Gastrointestinal Symptom Rating Scale (GSRS) was found to be applicable in terms of symptoms evaluation. Although limited, double-blind-placebo-controlled (DBPC) procedure remains to be the golden standard in NCGS investigation, yet, single-blinded procedure is allowed for the purposes of clinical practice [29,30,31]. The guidelines stress the importance of patient compliance, especially when it comes to shift to GFD, which should be discussed with a dietitian before implementation [29].

Back to the limitations—it is recommended to use gluten in the form of commonly consumed food products, during gluten challenge, rather than in the form of gluten capsules. Nevertheless, there is presumption that ATIs and FODMAPS—as the constituents of grains—interfere with the DPBC results [6,30,32]. Moreover, since the study on patients complaining about IBS-like symptoms, it was revealed that almost two-thirds of questioned patients presented nocebo effect after elimination diet, which seems to have same significant influence on performing DBPC during gluten challenge [29,33].

**Figure 3:**
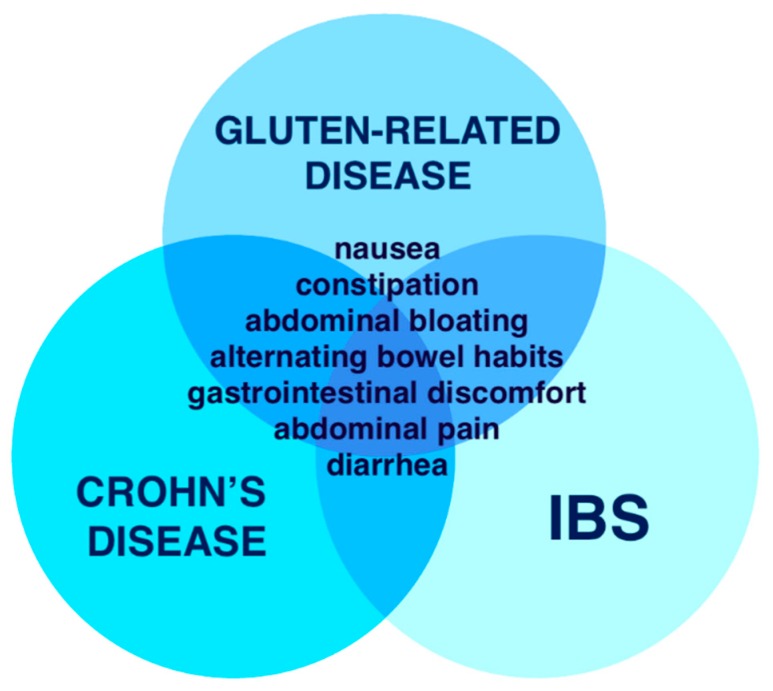
Overlapping symptoms in Crohn’s disease, IBS and gluten-related disease.

The fact that numerous symptoms manifested by active NCGS can be either vague or simply mimic other medical conditions, makes the diagnostic process long lasting and complex. For instance, bloating, abdominal pain, and irregular bowel movements are typical symptoms seen in IBS [20]. The overlapping symptoms of IBS, Crohn’s disease and GRD are shown in Figure 3.

The similarity between symptomatology of IBS and NCGS may lead to a wrong diagnosis and ineffective treatment [6]. The clinical case described by Vojdani and Perlmutter, presented a 49-year-old woman formerly diagnosed with IBS. The patient complained about abdominal pain, constipation, acid reflux and headache. Following conditions were contemplated and finally excluded: autoimmunological disorders, abnormal level of thyroid hormones, *H. pylori* infection [34]. Consequently, in the course of inappropriate therapy, the patient developed symptoms imitating systemic lupus erythematosus. Furthermore, the patient showed some improvement after corticosteroids administration, which appeared to be confusing for making right diagnosis as well [34]. Ultimately, after years of inappropriate treatment, the NCGS turned out to be the reason for patient’s affliction. In addition, a few studies indicate that NCGS can be primary trigger for developing IBS. Virtually, as NCGS and IBS-like symptoms tend to overlap, the diagnostic process is particularly challenging [20,35,36].

Even though the diagnosis within the wide spectrum of bowel diseases was made, in the case of continuous therapy failure, it is crucial to reconsider NCGS as the possible cause. A clinical case of a patient with NCGS overlapping Crohn’s disease has been reported. The onset of Crohn’s disease is characterized mainly by unspecific symptoms, including diarrhea, weight loss, right lower quadrant abdominal pain, which proceed in a gradual way, with very harmful effects [37]. In the above-mentioned case report, the patient suffered from refractory Crohn’s disease for 14 years and elevated IgG class antibodies directed against native gliadin (AGA) were detected, which shed a light on gluten related disorder. Introduction of GFD ceased diarrhea and enabled the patient to gain weight [34]. It is worth highlighting that NCGS patients are twice as likely to have AGA positivity [38].

At present, the linkage between gluten sensitivity, such as CD, and neurological disorders seems to be obvious. So far, numerous studies have unveiled extra intestinal symptoms affecting the peripheral and central nervous system due to celiac sprue. Although not fully understood yet, a wide range of NCGS neurological complications has been reported too. The state-of-the-art knowledge on NCGS revealed its association with transient and subtle cognitive impairment, being called “brain fog” [39]. Some scientists suggest NCGS to worsen symptoms in the context of depression but further examination must be performed to comprehend and determine NCGS relation with depressive disorders [32]. Busby et al. in their meta-analysis pointed out that standardization of methods measuring dietary adherence and mood symptoms is vital in terms of future research. Nevertheless, they admit that the gluten elimination diet may be an applicable treatment for mood disorders in patients suffering from gluten-related diseases [40].

It has not been until recently, when researchers explored that NCGS may be associated with gluten ataxia (GA), as the patients with typical GA symptoms did not meet criteria for CD diagnosis [41]. NCGS symptoms are believed to originate from an innate immune response. Interestingly, autoimmune diseases are reported to be more frequent in this group of patients, comparing to sheer IBS patients [42].

## 3. Results

Comparison of selected clinical trials concerning NCGS is shown in Table 2. In the study by Capannolo et al. patients with CD and WA were excluded while in the study by Elli et al. patients without CD, WA, IBS were enrolled. The prevalence of NCGS, CD and WA among patients with functional GI symptoms in the study of Capannolo et al. was estimated to be 6.88%, 6.63% and 0.51%. Capannolo et al. indicate that high frequency of visits due to gluten-related symptoms is not associated with high prevalence of GRDs. Ellie et al. established that 14% of patients, suspected to have NCGS because of responding to gluten withdrawal showed a symptomatic relapse during the gluten challenge. It was highlighted that GFD can have a beneficial effect even in the absence of CD or WA. However, there are certain limitations seen in both of compared above research papers. The research of Capannolo et al. was lacking blindness in GFD challenge and missing evaluation of possible influence of other food components. Besides it was conducted before Salerno Criteria were introduced (2015). A choice of timing and gluten dosage shown in the research of Elli et al. was not in line with the timing suggested by Salerno criteria. In addition, the protocol did not make use of a scheduled diet besides GFD. Moreover, a nocebo effect may be presumed, in consistence with symptomatic deterioration observed in the placebo group. Other diet variables in both studies cannot be excluded (ATIs) [43,44].

In 2015, Zanini et al. published a prospective, randomized, double-blind, placebo-controlled study on patients without CD or wheat allergy as seen in Table 2. Scientists observed 35 patients (31 females and 4 males) being on a GFD due to their own initiative because of gastrointestinal symptoms they had had on a diet containing gluten. They were switched to a diet containing gluten. Participants’ ability to distinguish between flours containing gluten and gluten-free was assessed, as well as their score in the Gastrointestinal Symptoms Rating Scale (GSRS). In order to participate in the study, patients had to be over 6 months a self-prescribed GFD and have a Gastrointestinal Symptoms Rating Scale (GSRS) below 4. The CD had had to be excluded before the start of the GFD. Before the beginning of the study t-TG antibody levels were measured and patients were instructed how to keep a diet diary. After 3 months, t-TG antibody level was checked again and GSRS questionnaire was performed. The participants received 10-g sachets containing gluten-free or gluten- containing flour labeled A or B. Patients were ordered to add contents to the pasta or soup for 10 days. Then for 2 weeks there was a washout period. Then, the patients received a second sachet with the other label, which they were to consume for 10 days. The primary outcome was the ability of the participants to correctly identify flour containing gluten. The study showed that only 34% (12 participants) correctly identified gluten- containing flour. Two thirds of the participants were not able to properly identify flour containing gluten. Almost half of the participants 17 (49%) misidentified gluten-free flour as gluten-containing flour, but those patients recorded symptoms and their GSRS scores increased on the flour not containing gluten. The gluten-free flour used in this test contained FODMAP [45].

Hollon et al. in their study (Table 2) disclosure ex-vivo gliadin effect on gut permeability in patients with active celiac disease (ACD), remission celiac disease (RCD) and gluten sensitivity (GS). The results of the research indicated that in all four groups, including control group (NG), there is certain response to gluten administration [46]. Researchers reported increased permeability particularly comparing ACD and GS groups to RCD, which is due to gluten induced alteration of intestinal barrier. Furthermore, researchers by means of quantification method investigated changes in following cytokines IL-6, IL-8, IFN-γ, TNF-α, which showed no significant difference, however, in this case a short period of incubation could implicate results. It should be emphasized that lack of blindness in GFD challenge while recruiting GS group along with lack of GFD challenge in the control group are important limitation in discussed study and could impact final results [46].

Shahbazkhani et al. investigated the relationship between dietary habits in IBS patients and consequent symptom fluctuations (Table 2). In particular researchers were interested in gluten impact on wellbeing of IBS patients and weather it may induce IBS-like symptoms. After rigid inclusion and exclusion criteria, strict six-week GFD 72 patients were recruited and divided into two groups: gluten containing group (study group), gluten free group (placebo group). Symptoms were analyzed by means of visual analogue scale (VAS). The results of the research revealed significant worsening of symptoms in a study group after gluten powder challenge. Scientists reported increase of overall symptoms such as satisfaction with stool consistency, tiredness, nausea, bloating in study group comparing to the control one. The results occurred to be statistically significant [47]. Nevertheless, there was limitation such as gluten form—a packet of 100 g powder, which is not recommended anymore by Salerno criteria [29].

According to the study published in Gastroenterology, scientists discovered that FODMAPs are another wheat antigen along with gluten triggering symptoms in patients with NCGS. Biesiekierski et al. conducted a double-blind crossover trial in which participated 37 patients suffering from NCGS and IBD. The following exclusion criteria were applied: age less than 16 years, CD confirmed by genetic tests and duodenal biopsy, alcohol abuse, chronic non-steroidal anti-inflammatory drugs (NSAIDs) and immunosuppressant treatment, uncontrolled psychiatric illness. Patients who had confirmed symptoms of IBS by accomplished the Rome III criteria and symptoms well controlled on a GFD were qualified for the study. Another requirement was to follow the GFD 6 weeks before this clinical trial. The first stage of the study was identical for all participants and the task was consuming for a one week a gluten-free and low FODMAPs diet. After a 2-week washout period, patients were randomly assigned to the three groups: high-gluten, low-gluten and placebo, without introducing FODMAP into the diet. The symptoms of the patients were measured by using 100-mm VAS scoring and Daily-Fatigue Impact Scale (D-FIS). All participants were asked to return to the second stage of this study—trial in which all patients received each diets for 3-days [48]. Gluten-specific responses were found only in 8% of patients. Scientists found a high nocebo effect and reproducibility of induction of symptoms in each arm was low [48].

Biesiekierski et al. noticed that patients with NCGS do not present a statistically significant occurrence of symptoms after introducing gluten into the diet, if at the same time they limit products rich in FODMAP (Table 2). These results may suggest that the symptoms in patients suffering from NCGS may in many cases be associated with intolerance to the contained sugars, but not hypersensitivity to gluten. Surprisingly, the patients involved into study evinced eminently high VAS ratings for their symptoms, despite being on GFD. Furthermore, an anticipatory nocebo response could influence the final results of this DBPC research. It is interesting that all participants eventually returned to GFD at the end of the trail as they ‘subjectively describe feeling better’ [48].

Scientists from Oslo, Skodje et al., conducted a study in which took part 59 patients on a GFD, in whom CD was excluded (Table 2). Participants were divided into three groups: receiving diet including gluten (5.7 g), fructans (2.1 g) and placebo. The clinical trial lasted 7 days and was preceded by a 1-week washout period. The following symptoms were recorded: pain, bloating, diarrhea, constipation, nausea, dizziness, weakness, sleepiness and tiredness. Participants filled a questionnaire containing 13 questions about their gastrointestinal symptoms and filled VAS. The results were measured by GSRS, Irritable Bowel Syndrome scale (GSRS-IBS), VAS, Short Form-36 (SF-36) and Giessen Subjective Complaint List [49]. Scientists observed that daily symptoms calculated using VAS score were significantly higher in fructans diet. Furthermore, they noticed that overall GSRS-IBS was higher in the FODMAPs group (38.6 g) than in the gluten group (33.1 g) and placebo (34.3 g). More ailments were recorded in the group receiving fructans, compered to two another groups. In addition, it was demonstrated that a diet rich in FODMAPSs caused greater weakness and decreased vitality compared to the placebo and gluten groups. The results of the study indicate that FODMAPs are a trigger factor of gastrointestinal complaints in patients suffering from NCGS [49]. Thus, scientists are leaning towards renaming NCGS to a more recent NCWS [27].

Di Sabatino et al. observed increased severity of intestinal symptoms (abdominal bloating, abdominal pain) and extra intestinal symptoms (foggy mind, depression, and aphthous stomatitis) among subjects with suspected NCGS (excluded CD and WA). Although, this study did not make a significant contribution in development of knowledge about NCGS and had some weaknesses such as lack of a control group, it indicates possible symptoms experienced by NCGS patients (Table 2) [50].

In order to prove that gluten is a trigger factor in patients with NCGS, Rosinach et al. conducted a study in which 18 participants were assigned to gluten or placebo groups. In 10 out of 11 patients, symptoms worsened in response to a gluten-containing diet, 7 of which were withdrawn from the study due to the severity of the symptoms [51]. There was no early termination in the placebo group although in 2 participants symptoms were observed (Table 2) [51].

Carroccio et al. collected and analyzed data from 200 patients examined in previous study with diagnosed NCWS. Their findings are interesting because about 90% of patients who maintained wheat-free diet (WFD) were characterized by significant improvement of IBS symptoms [52]. The authors came to the conclusion that NCWS is a persistent condition and patients with NCWS should therefore be correctly identified and treated with WFD (Table 2) [52].

Roncoroni et al. conducted a study on dietary exposure to different amounts of gluten in patients meeting the criteria of the NCGS [53]. Researchers observed different reactions of patients after the introduction of gluten. Some of them had a worsening of well-being and increased symptoms after a small dose of gluten, others observed this effect after the medium dose and others only after a high dose of gluten (Table 2) [53].

Carrocio et al. in their study in 2011 emphasize the link between particular food ingestion and deteriorating symptoms in a subgroup of IBS patients [54]. It clearly shows alleviation of the symptoms in 22% of IBS patients—whose previous treatment was ineffective—after eliminating gluten from the diet. Moreover, researchers excluded association of DQ2 and DQ8 haplotypes with frequent gluten sensitivity, however, patients presenting food hypersensitivity (FH) to both wheat- and cow’s milk-protein were reported to be often DQ2/DQ8 positive. Fecal eosinophil cationic protein (ECP) may be useful while identifying FH in IBS-patients (Table 3) [54].

Carroccio et al. in their study published in 2012 examined individuals with non-celiac WS, diagnosed by DBPC challenge with IBS-like symptoms, compared to CD patients and IBS patients [55]. Authors described presence of two types of WS subjects: WS similar to CD and WS associated with multiple food hypersensitivity. Besides, symptoms such as anemia, weight loss, self-reported wheat intolerance, coexistent atopy, and food allergy in infancy were noticed more often in WS compared to IBS controls. Furthermore, WS individuals were characterized by higher frequency of presence IgG/IgA anti-gliadin in serum, basophil activation (assessed by flow cytometric method) and histology specific eosinophil infiltration of the duodenal and colon mucosa. This study shows the differences between non-celiac WS and other gluten-related disorders (Table 3) [55].

Volta et al. in their study, assessed the level of immunoglobulin distinctive for CD in patients with GS comparing to CD [56]. They revealed that 50% of GS patients presented IgG AGA, whereas IgA AGA was seen only in a few patients in study group. Besides, researchers observed absence of IgA EmA, IgA tTGA, IgG DGP-AGA, which are typical for CD, within GS group (Table 3) [56].

Basing on a study group conducted by Volta et al., Caio et al. continued research on AGA IgG [38]. Scientists aimed to explore GFD impact on AGA IgG titer in AGA IgG positive patients (44 individuals) with NCGS. After six months of GFD AGA IgG disappeared in all the patients (Table 3).

Carrocio et al., in another research conducted in 2015, evaluated and described frequent ANA positivity within NCWS patients group [57]. The study demonstrated ANA positivity occurring along with DQ2/DQ8 haplotypes. As it was previously discussed, DQ2/DQ8 positivity is a distinctive feature of CD rather than NCWS. Thus, researchers highlight the need of intraepithelial intestinal flow cytometric pattern, which is an accurate method identifying seronegative CD patients, in the initial diagnostic biopsy. However, scientists found autoimmune diseases (AD) particularly frequent in study group. Autoimmune thyroiditis was reported to be the most frequent AD and amounted for 22% and 24% in retrospective and prospective groups respectively Table 3) [57].

Infantino et al. similarly to Volta observed frequent IgG AGA occurrence in NCGS patients, however, the author highlights that it is still lacking diagnostic accuracy. Nevertheless, in some cases, it can be helpful in the diagnostic process of NCGS patients [58].

Papers included in Table 3. Indicate IgG AGA and ECP to be helpful diagnostic tool while diagnosing NCGS. Still they have limited application in a large group of NCGS patients and cannot be widely used in NCGS diagnostic protocol [54,58].

## 4. Discussion

Nowadays, a gluten-free diet is fashionable and is promoted by many celebrities. Many people undergo this fashion and despite lack of symptoms, try to reject gluten because they believe it may harm their health. In 2016, as much as USD 15.5 billion was spent on gluten-free food sales. This value is more than twice as high as in 2011. Lack of gluten in food consumed by people who tolerate it well may not bring favorable results.

In a study conducted by Norsa et al., children with CD were tested for at least one year on a GFD diet. As many as 34.8% of children on GFD diet had high concentrations of triglycerides on fasting, 24.1% high concentration of LDL cholesterol and 29.4% increased blood pressure. In 52 out of 114 participants there were available cards with information on blood lipids concentration before GFD introduction. 24% of children on GFD had had LDL cholesterol borderline values. That was much more than before the introduction of the diet (10%). However, these data did not meet the value of statistical significance (*p* = 0.09) [59].

Studies show that gluten may have a positive effect on triglyceride levels. In a clinical trial in which 20 adults with hyperlipidemia took part, a group with a balanced diet and a group with a high gluten content (78 g per day with an average human intake of 10–15 g) were studied. The high gluten diet group had a decreased triglyceride concentration of 19.2% (*p* = 0.0003) compared to the control group after one month of the study [60]. In another study, a group of patients consuming 60 g of gluten per day had a 13% (*p* = 0.05) lower triglyceride concentration compared to the control group [61]. In a study published in 2017, the estimated gluten consumption lead to the protective effect against cardiovascular disease (HR 0.85, 95% CI 0.77-0.93, *p* = 0.002) [62].

Gluten-free products can also be more than twice as expensive as regular products [63]. There are other disadvantages of GFD. The GFD turned out to be poor in trace elements and vitamins, such as zinc, iron, magnesium, calcium, vitamin D, vitamin B_12_, folate, and fiber [64,65]. Furthermore, Tovoli et. al. compared scores obtained by NCWS and CD individuals using quality of life questionnaire (CDQ) before GFD introduction and after at least one year. NCWS patients still reported intestinal and parenteral symptoms, although symptoms were significantly reduced in comparison to period before GFD. Therefore, other factors influencing NCWS should be investigated [66].

Finally, based on revised research results, it is clear that NCGS still remains to be the subject of uncertainty, especially in terms of other wheat components contribution to its symptoms. There are only a few published forms of research in the last six years. It should be stressed that it is hard to compare the results of each study as obtained methods and criteria significantly vary. Moreover, the timing of onset of each research was of a great importance as some of them were conducted before Salerno criteria were introduced, which led to many interpretations and qualification protocols of patients with NCGS-like symptoms. Further investigations and seeking for biomarkers would play key role in improving of the diagnostic process and patients’ follow up.

## 5. Conclusions

1. Symptoms of non-celiac gluten sensitivity are similar to gluten-related disease, irritable bowel syndrome and Crohn’s disease.
2. With Salerno Experts’ Criteria of non-celiac gluten sensitivity it is possible to diagnose patients properly and give them advice about nutritional treatment.

## Figures and Tables

**Table 1:** Comparison of prevalence, pathogenic, and diagnostic features of gluten related disorders (GRDs); non-celiac gluten sensitivity (NCGS), IgA anti-EMA (IgA antibodies against endomysium), IgA anti-tTG (IgA antibodies against transglutaminase), IgG anti-DGP (IgG antibodies against deamidated gliadin peptides).

	Celiac Disease	NCGS	Wheat Allergy
Prevalence	0.5–1.7%	no population studies	0.5–9% in children
Pathogenesis	autoimmune	non-specific immune response	IgE mediated response
DQ2-DQ8 HLA haplotypes	positive in 95% cases	positive in 50% cases	negative
Serological markers	IgA anti-EMA, IgA anti-tTG, IgG anti-DGP, IgA anti-gliadin	IgA/IgG anti-gliadin in 50% cases	specific IgE antibodies against wheat and gliadin
Duodenal biopsy *	Marsh I to IV with domination of Marsh III and IV	Marsh 0-II, but according to some experts Marsh III might also be in NCGS	Marsh 0-II
Duodenal villi atrophy	present	absent	might be present or absent

* Marsh classification.

**Table 2:** Comparison of selected researches on NCGS.

References	Study Group	Exclusion Criteria	Methods	Findings	Comments
Biesiekierski et al. 2013	IBS patients fulfilling Rome III criteria in NCGS criteria, on GFD for 6 weeks	CD, IBD, age < 16, serious GI disease (cirrhosis), psychiatric disorders, alcohol abuse, NSAIDs and immunosuppressive treatment	GFD for 6 weeks, next 2-weeks diet low in FODMAPs, then 3 days one of the groups—high gluten 16 g, low gluten 2 g gluten or 14 g whey protein, control for 2 weeks washout period and crossover to another group for 3 days. **The primary outcome:** GI symptoms measured by using 100-mm VAS scoring. **The secondary outcome:** Fatigue measured by Daily-Fatigue Impact Scale (D-FIS), gliadin-specific T-cell response, biomarkers.	**The primary outcome:** Gluten-specific responses only in 8% of patients, 16% had worsening of overall GI symptoms in high gluten diet	**Limitations:** The nocebo effect was present independent of substances which was delivered
				**The secondary outcome:** Fatigue measured by D-FIS was lower in the low FOTMAPs diet, no significant difference in biomarkers, physical activity or sleep was observed, only one patient had gliadin-specific T-cell response.	
Capannolo et al. 2014	Individuals with gluten related symptoms	CD and WA	**NCGS finding:** on the basis of the disappearance of the symptoms within GFD 6 month, followed by 1month GD.	**CD** patients: 26 (6.63%); **WA** patients: 2 (0.51%); **NCGS** patients: 27 (6.88%). Patients with **no change of symptoms** after GFD 337 (85.9%)	**Limitations:** Lack of blindness in GFD challenge
					Missing evaluation of possible influence by other food components
				**Symptoms in 74% NCGS patients:**	
				**Intestinal:** abdominal pain, diarrhea, constipation, alternating bowel function, epigastric pain	
				**Extra intestinal:** malaise, chronic fatigue, headache, anxiety, confused mind, depression, joint/muscle pain, resembling fibromyalgia, weight loss, anemia, dermatitis, rash	
				**Related disorders in NCGS patients:** lactose intolerance, autoimmune thyroiditis, type 1 diabetes, psoriasis, sarcoidosis	
Zanini et al. 2015	Individuals on gluten-free diet (GFD) on their own initiative	CD, non-strict adherence to a GFD, symptomatic on GFD	The primary outcome: the ability of the participants to correctly identify flour containing gluten. GSRS questionnaire was performed	Only 34% (12 participants) correctly identified gluten- containing flour fulfilling the clinical diagnostic criteria for NCGS.	The gluten-free flour used in this test contained FODMAP
Hollon et al. 2015	Individuals with Active CD, CD in Remission, Gluten Sensitivity (GS)	Positive CD serology, abnormal duodenal histopathology, unresponsive to gluten open challenge	**GS finding:** on the basis of the disappearance of the symptoms within GFD; non-blinded gluten challenge (10 g) for a minimum of 2 months before endoscopy	Increase of gut permeability after PT-gliadin ex-vivo administration in all three study groups, and in control group	**Limitations:** Lack of blindness in GFD challenge—possible placebo-response; Lack of GFD challenge in the control group—possible individuals with undiagnosed GS/CD
Shahbazkhani et al. 2015	Individuals with newly diagnosed IBS based on the Rome III criteria	Patients with CD, GFD introduced ever in medical history, self-exclusion of wheat from the diet, IBD, diabetes, concurrent drugs for depression/anxiety, NSAI drugs intake, abnormal levels of: glucose, urea, creatinine, sodium, potassium, hemoglobin, erythrocyte sedimentation rate, thyroid function tests	**GS finding:** IBS diagnosed patients responding to gluten challenge by means of statistically significant worsening of symptoms after gluten meal packet. Patients previously following strict GFD, continued gluten challenge for 6 weeks	Significant increase for following symptoms after gluten-containing meal challenge: bloating, abdominal pain, stool consistence, tiredness, nausea	**Limitations:** Packets containing gluten meal in the form of powder—not recommended according to Salerno criteria
					**Pros:** double-blind randomized placebo-controlled trial
Di Sabatino et al. 2015	Suspected NCGS individuals	CD, WA	Individuals were randomly assigned to groups given gluten or placebo for 1 week, each via gastro-soluble capsules. After a 1 week of gluten-free diet, participants crossed over to the other group.	Gluten group: significantly increased overall symptoms (intestinal symptoms: abdominal bloating and pain, extra-intestinal symptoms: foggy mind, depression, aphthous stomatitis) vs. placebo group.	**Limitation:** small study group
Elli et al. 2016	Individuals with functional gastroenterological symptoms with enrolled on 3-week-long GFD	CD, WA, IBS psychiatric disorders, major abdominal surgery, diabetes mellitus, systemic autoimmune diseases, previous anaphylactic episodes, any systemic disorders, pregnant, breast feeding women, GFD in previous 6 months, patients on pharmacological therapy	**Phase 1.** GFD response individuals: questionnaire and next 3-week GFD. Patients with significantly improvement carried on to next phase. **Phase 2. 98 subjects.** GFD response patients—maintain strict GFD and underwent placebo-controlled double-blind gluten challenge with crossover. Patients were randomized to take gluten in capsules or placebo (rice starch) for 7 days. Total duration: 21 days: 7 days on gluten or placebo, 7 days wash-out, 7 days on gluten or placebo.	28 individuals from phase 2 reported a symptomatic relapse and deterioration of quality of life. 14 patients responded to the placebo ingestion. About 14 patients responding to gluten withdrawal showed a symptomatic relapse during the gluten challenge—they are suspected to have NCGS.	**Strengths:** The blinding of patients and doctors, and the crossover design.
					**Weaknesses:** arbitrary choice of timing and gluten dosage, the protocol did not make use of a scheduled diet besides GFD, other diet variables cannot be excluded (ATIs). Symptomatic deterioration was also observed in placebo group.
Rosinach et al. 2016	Individuals with clinical GI symptoms and clinical and histological remission after GFD	Age < 18, CD, NSAIDs and Olmesartan immunosuppressive treatment in last month, immunosuppressive therapy, parasitic or H. pylori infection, AD, pregnant or breastfeeding women, participation in other randomized controlled trials in the last 4 weeks, serious GI diseases and GI surgery, severe comorbidities, failure to comply with the protocol requirements	Patients were randomly assigned to gluten group (20 g/day, n = 11) and placebo (n = 7). Clinical symptoms were measured by VAS, quality of life using GIQLI. Scientists examined the presence of gamma/delta+ cells and transglutaminase deposits.	91% of patients with clinical relapse during gluten challenge compared to 28.5% after placebo. Worsening results in clinical scores and GIQLI was observed in patients on gluten diet, but not in the placebo	**Limitations:** a small study group
			primary end-point: disease relapse after 6 months		
Carroccio et al. 2017	Induviduals with NCWS		Data collecting from a previous study of NCWS.	88% subjects improved after a diagnosis of NCWS; 145 of 148 patients on strict GFD (98%) had reduced symptoms, compared to 30 of 52 patients who was not on GFD, 20 (from 22) subjects who repeated DBPC challenge reacted to wheat. NCWS is a persistent condition.	**Limitations:** not thoroughly discussed exclusion criteria
Skodje et al. 2018	Individuals self-reported NCGS on gluten-free diet (GFD) on their own initiative for at least 6 months	**Exclusion criteria: CD, WA, IBD,** gastrointestinal comorbidity, allergy to nuts and sesame, alcohol abuse, pregnancy, breast feeding, women in fertile age without using contraception, long travel distance, considerable infection, patients on immunosuppressive agents’ therapy	GFD for 6 months, next 7 days on one of three diets challenge (gluten 5,7g, fructans 2,1g and placebo), 7 days washout, then crossover to next diet. **The primary outcome:** gastrointestinal symptoms measured by GSRS-IBS. **The secondary outcome:** daily GI symptoms measured by VAS, life quality depends on symptoms by SF-36, depression and anxiety symptoms measured by Hospital Anxiety and Depression Scale, Fatigue measured by Giessen Subjective Complaint List and VAS	**The primary outcome:** overall GSRS-IBS higher in the fructans group (38,6) than in the gluten group (33,1) and placebo (34,3). **The secondary outcome:** overall GI symptoms measured by VAS higher in FODMAPs diet, decreased vitality and greater weakness in the group of patients receiving fructans	**Limitations:** high nocebo response
Roncoroni et al. 2019	Individuals with NCGS criteria, complaining about functional GI symptoms	CD, WA, IBD, adult age (<18 years old), positive anti-tissue transglutaminase IgA, psychiatric disorders, major abdominal surgery, diabetes, GFD for previous six months, autoimmune diseases and systemic disorders, pregnancy, breast feeding, experience of anaphylaxis and patients during pharmacotherapy	GFD for 3 weeks, then exposure to diets with gradually increasing the amount of gluten: low-gluten diet (3.5–4 g gluten/day, week 1, n = 22 + 2 dropped out patients), mid-gluten diet (6.7–8 g gluten/day, week 2, n = 14), and a high-gluten diet (10–13 g gluten/day, week 3, n = 8). Patients without GI symptoms on a previous diet were classified into more gluten- containing diet. Patients with GI symptoms were shifted back to a well-tolerated diet. Daily GI symptoms measured by VAS, life quality depends on symptoms by SF-36	Different reactions of patients after the introduction of gluten.	Limitation: a small study group

**Table 3:** Researches on potential NCGS biomarkers.

References	Study Group	Exclusion Criteria	Methods	Findings	Comments
Carroccio et al. 2011	Individuals who fulfilled Rome II criteria for IBS	Individuals with organic diseases	Symptom severity questionnaire was analyzed, fecal samples were assayed, and levels of specific immunoglobulin E were measured. Patients were observed for 4 weeks, placed on an elimination diet (without cow’s milk and derivatives, wheat, egg, tomato, and chocolate) for 4 weeks, and kept a diet diary. Those who reported improvements after the elimination diet period were then diagnosed with food hypersensitivity (FH), based on the results of a double-blind, placebo-controlled, oral food challenge (with cow’s milk proteins and then with wheat proteins).	40 of patients with IBS (25%) were found to have FH. Levels of fecal ECP and tryptase were significantly higher among patients with IBS and FH than those without FH. The ECP assay was the most accurate assay for diagnosis of FH, showing 65% sensitivity and 91% specificity.	Limitations: recruitment of patients not in line with Salerno criteria.
Carroccio et al. 2012	Individuals with non-celiac wheat sensitivity (NCWS),	IgA deficiency, self-exclusion of wheat from the diet, lack of DBPC-challenge method in the diagnosis	A review of the clinical charts of patients with IBS-like presentation, diagnosed with WS challenge in the years 2001-2011.	1/3 IBS patients who underwent DBPC wheat challenge were really suffering from WS. WS group: higher frequency of anemia, weight loss, self-reported wheat intolerance, coexistent atopy, and food allergy in infancy than the IBS controls, higher frequency of positive serum assays for IgG/IgA anti-gliadin and cytometric basophil activation in “in vitro” assay, eosinophil infiltration of the duodenal and colon mucosa. Two groups with distinct clinical characteristics were identified: WS alone (with similar to CD clinical features) and WS with multiple food hypersensitivity (clinical features similar to those found in allergic patients)	Limitations: recruitment of patients not in line with Salerno criteria.
Volta et al. 2012	Individuals with GS (NCGS)	CD, WA	Retrospective evaluation of collected samples from GS (study group) and CD (control group) individuals. Assessment of IgG/IgA AGA, IgA EmA, IgA tTGA, IgG DGP-AGA. HLA DQ2/DQ8 presence was assessed	GS is characterized by IgG AGA positivity (50%), although is less common comparing to CD. IgA AGA are rare. GS patients were lacking EmA, tTGA, and DGP-AGA.	Limitations: not thoroughly described exclusion criteria for study group
Caio et al. 2014	Individuals with NCGS with simultaneous AGA IgG positivity	CD, WA	AGA of both IgG and IgA classes were assayed by ELISA in 44 NCGS and 40 CD patients after 6 months of gluten-free diet.	AGA IgG in NCGS patients disappear after introduction of GFD.	
Carroccio et al. 2015	NCWS patients of the retrospective cohort study	Incomplete clinical charts were excluded from retrospective study; for both studies: EmA in the culture medium of the duodenal biopsies, self-exclusion of wheat from the diet and refusal to reintroduce it before entering the study, other organic gastrointestinal diseases.	NCWS patients—tTG IgG, EmA IgA and IgG negative, absence of intestinal villous atrophy and WA. Patient medical records were reviewed to identify those with autoimmune disease (AD). CD or IBS served as controls. Serum samples were collected from all subjects and ANA levels were measured by immunofluorescence analysis. Participants completed a questionnaire and their medical records were reviewed to identify those with ADs. Individuals were randomly assigned to groups given gluten or placebo for 1 week, each via gastro-soluble capsules. After a 1 week of gluten-free diet, participants crossed over to the other group.	Patients with NCWS were more likely to be ANA positive than both patients with CD and IBS, in both the retrospective and prospective studies. Patients with NCWS showed a frequency of AD similar to CD, but significantly higher than IBS controls, in both the retrospective and prospective studies. NCWS or CD are more likely to be ANA-positive, have DQ2/DQ8 haplotypes and AD compared with patients with IBS.	Limitations: selection bias of the tertiary centers conducting research; evaluation of the duodenal histology; not in line with Salerno criteria
	NCWS patients of the prospective study				
Infantino et al. 2015	Induviduals with suspected NCGS	CD, WA	Evaluation of collected samples from GS (study group), CD and healthy (control group) individuals. Assessment of IgG/IgA AGA, IgA EmA, IgA tTGA, IgG/IgA DGP-AGA. HLA DQ2/DQ8 presence was assessed	Statistically significant correlation between AGA IgG and NCGS were found. However, AGA IgG still remains to be weak NCGS marker.	Limitations: recruitment of patients not in line with Salerno criteria; small study group
